# Clinical effect of a novel transpedicular reducer for reduction and bone grafting combined with pedicle screw fixation for thoracolumbar burst fractures

**DOI:** 10.1186/s12891-021-04423-1

**Published:** 2021-06-14

**Authors:** Menghan Cai, Zhijun Xin, Weijun Kong, Qian Du, Wenjun Ji, Fujun Wu, Jin Li, Jialin He, Wenbo Liao

**Affiliations:** Department of Spinal Surgery, The Affiliated Hospital of Zunyi Medical University, 149 Dalian Road, Huichuan District, Zunyi, 563099 Guizhou China

**Keywords:** Burst fracture, Thoracolumbar spine, Transpedicular reducer, Allogeneic bone, Spine surgery

## Abstract

**Background:**

Short-segment transpedicular screw fixation is a common method for the treatment of thoracolumbar burst fractures (TBFs),but this technique has many problems. Therefore,the purpose of this article is to observe and evaluate the clinical efficacy of a novel transpedicular reducer that we designed for fractured vertebral body reduction and bone grafting in the treatment of TBFs.

**Methods:**

From July 2018 to November 2020, 70 cases of TBFs were included. Thirty-five patients were treated with the novel transpedicular reducer for reduction and bone grafting combined with pedicle screw fixation (observation group), and 35 patients were treated with short-segment transpedicular screw fixation (control group). Before the operation, after reduction, and 3 days, 3 months,and 12 months after the operation, the two groups were assessed, and compared with respect to the anterior and middle heights of the injured vertebrae, the ratios of the anterior and middle heights of the injured vertebral body to the respective heights of the adjacent uninjured vertebral bodies (AVBHr and MVBHr, respectively), and the Cobb angle of the patients. We compared the pain VAS score and quality of life GQOL-74 score at the last follow-up. Finally,we evaluated the distribution of bone grafts and bone healing 12 months after the operation.

**Results:**

**T**he anterior height, middle height, AVBHr, MVBHr, and Cobb angle of the injured vertebral body in the observation after reduction, and 3 days, 3 months and 12 months post-operatively were compared with those of the injured vertebral body before operation. All of these parameters were improved, and the difference was statistically significant (*p* < 0.05). These parameters in the observation group at the above time points were significantly better than thoes in the control group at the corresponding time points (*p* < 0.05). The VAS scores at the last follow-up were significantly better than those of the control group (*p* < 0.05), but the GQOL-74 score differences were not statistically significant (*p* > 0.05). The observation group showed no obvious defects on CT at 12 months after the operation, and the bone healing was good.

**Conclusion:**

The novel transpedicular reducer for reduction and bone grafting combined with pedicle screw fixation for TBFs has good clinical efficacy.

## Background

Thoracolumbar burst fractures (TBFs) are among of the most common forms of spinal trauma in clinical practice. The axial compressive force on the centre column typically collapses the bone and causes the front and centre support columns to fail. Burst fracture subsequently occurs [1]. Most of these fractures (70%) occur at the thoracolumbar junction (Th11-L2) [2]. In young patients, falls from heights, traffic accidents, and sports injuries are the most common causes of vertebral fractures [2]. Simple falls are the most common cause of incomplete burst fractures in the elderly [3]. In the AOSpine thoracolumbar spine injury classification system, burst fractures are classified as A3 or A4 based on whether one or two end plates are damaged (A3 involves a single endplate fracture of the posterior wall of the vertebral body; A4 involves the upper and lower endplates and posterior wall of the vertebral body) [4]. These injuries can be devastating, and include paralysis, pain, deformity and loss of function [5–8].

The purpose of TBF treatment is to stabilize the spine, prevent short- and long-term deformities, prevent neurological decline, and improve clinical outcomes [9]. The stability of the spine is an important factor in determining the treatment method. Tezer et al. [10] believe that conservative treatment can be considered only if the nerve tissue and the posterior ligament complex are not damaged; howerver, a longer immobilization time may be required, and restoration of the normal sagittal position may fail [11]. Rapid surgery not only restores the sagittal position more reliably in some cases [12–14] but also restores nerve function more effectively to facilitate a more rapid recovery.

Clinically, the most widely used surgical method for the treatment of TBFs is short-segment fixation with pedicle screws. Because of its simple operation, it can effectively restore the height of the vertebral body and correct kyphosis. However, this operation causes substantial surgical trauma,, substantial intraoperative blood loss, and slow postoperative recovery and is prone to loss of correction and failure of internal fixation after surgery [15, 16].

To restore the height of the injured vertebrae, correct kyphosis, and promote healing of the injured vertebrae, we designed a novel transpedicular reducer according to the anatomical nature of the thoracic and lumbar pedicles, which can better restore the height of the vertebral body by manipulating the mechanical force. In addition, we intraoperatively implanted allogeneic bone. We compared and studied the clinical results of 35 cases with TBFs treated with the novel transpedicular reducer for reduction and bone grafting combined with pedicle screw fixation and 35 cases with TBFs treated with short-segment transpedicular screw fixation alone at the First Affiliated Hospital of Zunyi Medical University from July 2018 to November 2020.

## Method

### Inclusion criteria

The following inclusion criteria were employed in this study:(1) vertebral burst fracture confirmed by X-ray examination or CT scan; (2) AO classification as A3 or A4 burst fracture;(3) DEXA result t > − 2.5;(4) no vertebral body tumours; (5) no symptoms of nerve damage;(6) no serious heart, brain or lung problems; (7) no surgical contraindications, such as infections and blood clotting disorders; and (8) no history of thoracolumbar surgery.

### Clinical data

From July 2018 to November 2020, 70 patients with TBFs met the eligibility criteria for the use of posterior short-segment pedicle screw internal fixation and distraction reduction surgery. Thirty-five surgeries were performed with the novel transpedicular reducer for reduction and bone grafting combined with pedicle screw fixation (observation group) and 35 surgeries were performed with short-segment transpedicular screw fixation alone (control group) (see Table 1). The posterior wall of the vertebral body remained largely intact. No neurological deficit was observed.

**Table 1:** General information

	surgical sitesN	observation group35	control group35	*P*-value
	Age(year)	47.2±2.32	46.3±1.89	0.852
Gender	Male(n)	23	20	0.461
	Fmale(n)	12	15	
Fracture location	T11(n)	3	2	0.949
	T12(n)	11	10	
	L1(n)	17	19	
	L2(n)	4	4	
AOSpine classification	TypeA3(n)	25	24	1.000
	TypeA4(n)	10	11	
Causes of fractures	traffic accidents(n)	14	13	0.806
	falling from height(n)	21	22	
	operation time(min)	120.3±22.6	78.2±19.4	0.000*
complicatiocns	urinary retention(n)	1	3	0.081
	dural tear(n)	0	1	
	fluid leakage(n)	0	1	

*The difference between groups is statistically significant.*p* < 0.05

### Novel transpedicular reducer

#### Structural design

**Fig. 1:**
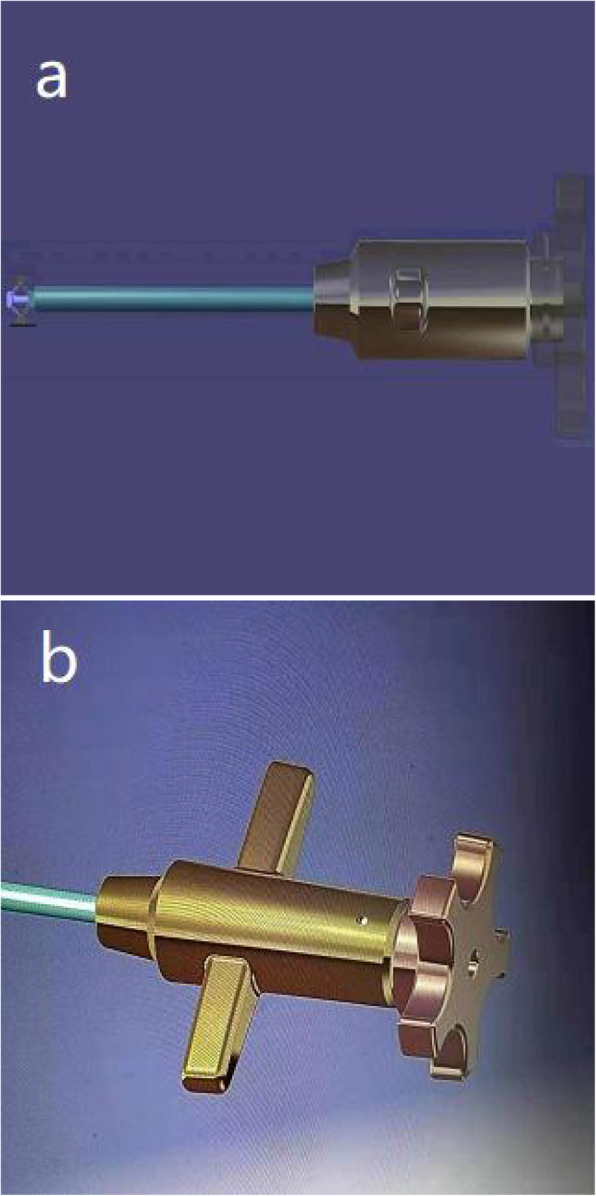
**a,b:**Structural diagram of the novel type of transpedicular reducer

A novel transpedicular reducer (Chinese patent number: ZL 2019 21,561,649.5) (as shown in Fig. 1) was designed. The reducer is composed of a knob, a fixed sleeve, a movable pull rod and two supporting plates. During operation, one hand grips the handle of the fixed sleeve, and the other hand rotates the knob, thereby driving the movable pull rod to move and drive the two supporting plates to open or close. The overall length of the reducer is 27.50 mm, the diameter in the closed state is 5.0 mm, the maximum diameter is 13.0 mm, and the expansion range is 8.0 mm.

#### Operational technical points

**Fig. 2:**
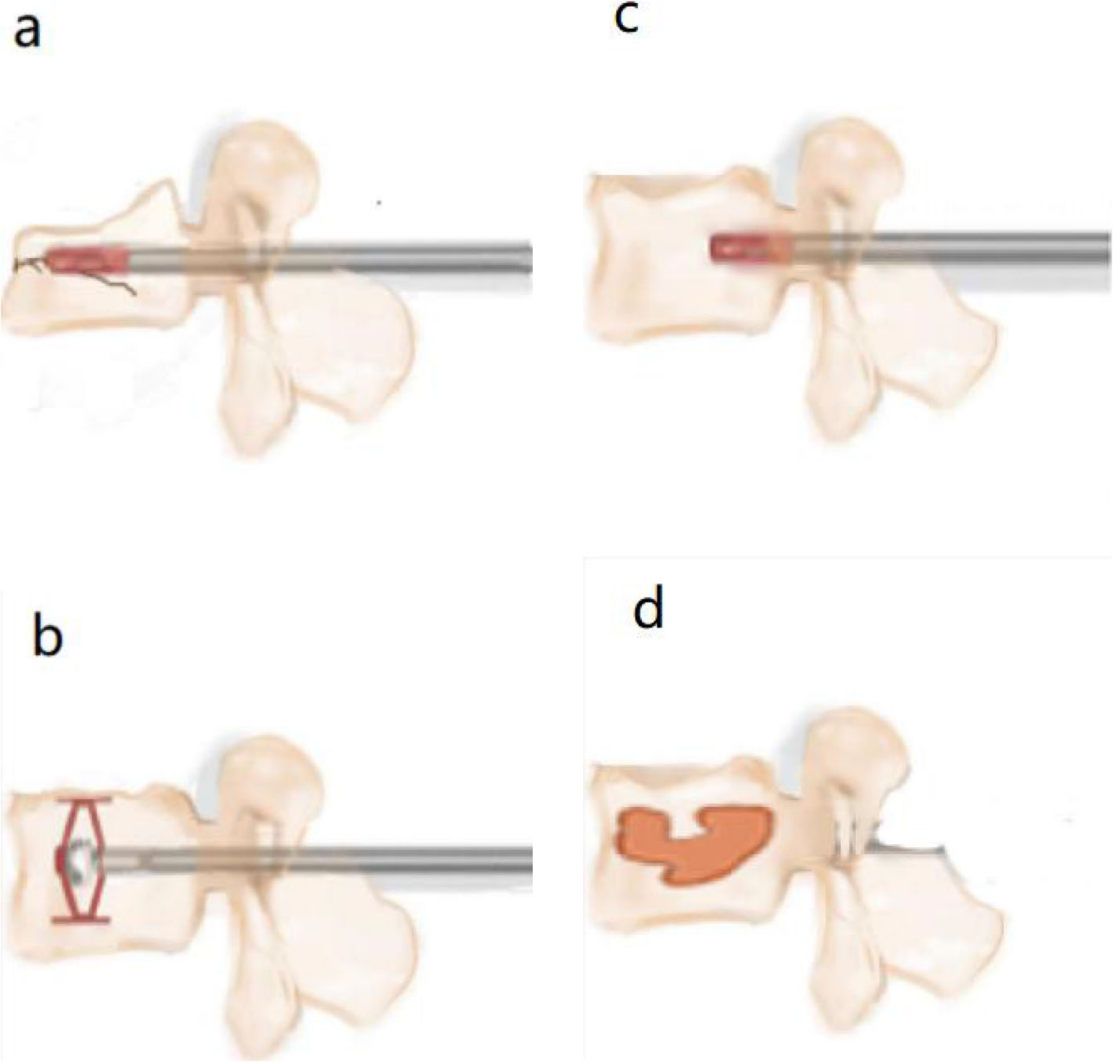
Schematic diagram of the strategic design for the application of the novel transpedicular reducer for a vertebral burst fracture. **a**:The novel transpedicular reducer enters the fracture through the pedicle; **b**: Open reduction is performed; **c**: The novel transpedicular reducer closes and exits the injured vertebra; and d: The novel transpedicular reducer can provide vertebral space for bone grafting of injured vertebrae

First, a path through the pedicle is established to reach the centre of the fractured vertebral body, and the novel transpedicular reducer is implanted in its unexpanded state through the path to make it reach the proper position in the vertebral body. Then, the knob is turned to expand the reducer and make it burst. The fractured vertebral body restores its height and shape and finally rotates the knob to close the reducer to withdraw from the vertebral body (Fig. 2).

### Operation

All patients were intubated under general anaesthesia with bent hips, bent knees, bowed waist and a prone position on the operating bed as well as with moderate overextension of the trunk to obtain better restoration of the injured vertebra. The two iliac abdominal regions were placed on cushioned support for fixation, and the abdominal regions were suspended to reduce abdominal pressure and intraspinal venous plexus bleeding. Regular disinfection was employed, and sterile towels were spread slightly laterally on bilateral marked points at approximately 0.5 cm. Needle biopsy was applied to the pedicle centre under the C arm with an oblique perspective to adjust the needle position and direction satisfactorily after needle positioning was fixed to guide the needle puncture point in the centre-cut skin and subcutaneous tissue. The fascia was cut with a high frequency electric knife followed by periosteal stripping on both sides of the shaft, electric coagulation and gauze tamponade haemostasis, which revealed the spinal segment, vertebra, and articular process. On both sides of vertebral pedicle, the positioning needle was placed into the needle point from the perspective of the C arm machine. The position and direction of the satisfactorily enlarged hole was adjusted from the vertebral segment. Four pedicle screws with a suitable diameter and length were installed, and C arm fluoroscopy was used to ensure that the pedicle screw position and direction were satisfactory. After the needle was used to place the pedicle into the needle point and after C arm fluoroscopy revealed that the location was accurate, the injured vertebral pedicle was used to establish an open channel. Under the guidance of the C-arm, the novel transpedicular reducer was implanted through the pedicle in an unexpanded state so that it could reach the proper position in the vertebral body. Then, the knob was expanded to restore the height and shape of the fractured vertebral body, and finally, the knob was turned close to the exit of the vertebral body The defect was filled with allogeneic bone granules implanted into the vertebra through the bone graft channel of the injured vertebra. Prebent titanium rods were installed and properly propped and fixed. C-arm fluoroscopy showed that the pedicle screw position and direction, as well as those of the injured vertebral body, were satisfactory. Next, the horizontal connecting rod was installed. A large amount of normal saline was used to wash the wound surface. No active bleeding was detected, and the gelatine sponge was covered. After the dressing, the instruments and brain cotton were assessed, a negative-pressure drainage tube was placed, the incision was closed layer by layer, a sterile dressing was wrapped and fixed, and the operation was completed.

### Patient evaluation

#### Measurement

Two radiologists obtained data on the injured thoracolumbar vertebrae before the operation; after reduction, and 3 days, 3 months and 12 months after the operation, the average value was used as the final value.

**Fig. 3:**
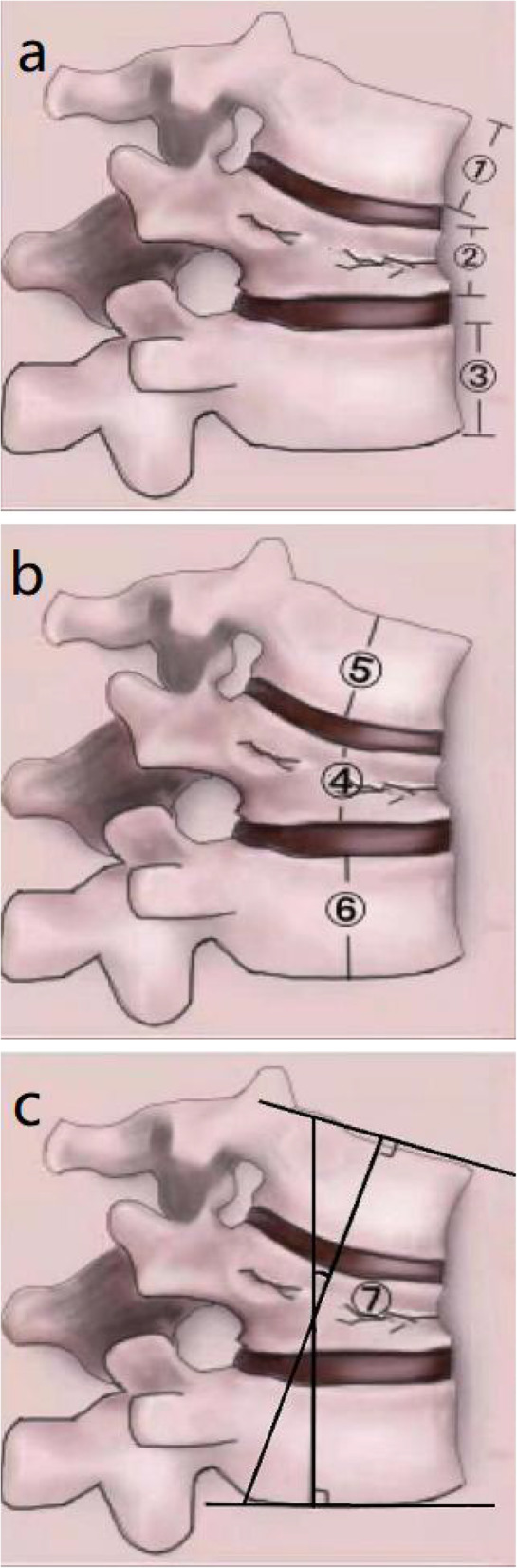
Measurement of the injured vertebral body. **a**: ② The injured vertebral anterior height; AVBHr = ②/[(① + ③) × 0.5]; **b**: ④ The injured vertebral medium height, MVBHr = ④/[(⑤ + ⑥) × 0.5]; **c**: ⑦ Cobb angle

- The anterior height and ratio of the injured vertebrae (AVBHr) (Fig. 3a)
- The middle height and ratio of the injured vertebrae (MVBHr) (Fig. 3b)
- The cobb angle of the injured vertebrae (Fig. 3c)

#### Observation

The pain VAS score and the quality of life GQOL-74 score of the two groups at the last follow-up were compared. The quality of life GQOL-74 scale is divided into four dimensions: psychological function, social function, physical function, and material life. The maximum score for each dimension is 100 points, yielding a total possible score of 400 points. The higher the score, the better the patient’s quality of life. We evaluated the distribution of bone grafts and bone healing 12 months after the operation.

## Statistical analysis

SPSS 18.0 statistical software was used for statistical analysis. First, the data were assessed to determine whether they conformed to a normal distribution. The VAS score, GQOL-74 score, anterior height, middle height, AVBHr, MVBHr, Cobb angle of the injured vertebrae, operative time and age were normally distributed data, expressed as the mean ± standard deviation (^−^x ± s). Immediately after reduction and 3 days, 3 months and 12 months after surgery, the anterior height, middle height, AVBHr, MVBHr, and Cobb angle of the injured vertebrae in the observation group were compared with those in the same group before surgery by repeated measures analysis of variance. If the difference was statistically significant, the independent-samples t test was used to compare the data in the same time period between the observation and control groups. The VAS and GQOL-74 scores of the two groups at the last follow-up were ranked data, and the rank sum test was used in the analysis. The independent-samples t test was used to analyse the age and operative time of the two groups. The chi-square test was used to analyse the sex, fracture site, AO spinal classification of the fracture vertebral body, fracture causes and complications of the two groups. *p* < 0.05 was considered statistically significant.

## Results

### Perioperative situation

All subjects successfully underwent the novel transpedicular reducer procedure for reduction and bone grafting combined with pedicle screw fixation (observation group) or short-segment transpedicular screw fixation alone (control group) and were followed up for more than 12 months. The mean operative time of the observation group was 120.3 ± 22.6 min, and that of the control group was 78.2 ± 19.4 min. No wound infection occurred post-operatively. In the observation group, only 1 patient (2.9%) had urinary retention, which was cured after symptomatic treatment; in the control group,5 patients (14.3%) had complications,(1 case of cerebrospinal fluid leakage, 1 case of dural tear, and 3 cases of urinary retention), and healed after symptomatic treatment. The incidence of complications in the observation group was lower than that in the control group, but the difference was not statistically significant (*p* > 0.05) (Table 2). The patients became active 2 to 3 days after bed rest.

**Table 2:** The anterior and middle heights, AVBHr, MVBHr and the Cobb angle of injured vertebrae pre- and post-operatively in the observation group

	Before the operation	After reduction	*P*-value^a^	3 days after the operation	*P*-value^b^	3 months after the operation	*P*-value^c^	12 months after the operation	*P*-value^d^
anterior height (mm)	20.56 ± 3.74	29.53 ± 2.53	0.000*	29.88 ± 2.52	0.000*	28.36 ± 1.93	0.000*	27.96 ± 0.72	0.001*
middle height (mm)	20.36 ± 4.20	27.54 ± 1.00	0.000*	27.70 ± 2.01	0.000*	27.05 ± 2.45	0.000*	26.83 ± 2.45	0.001*
AVBHr (%)	63.09 ± 8.91	97.22 ± 7.38	0.000*	97.54 ± 5.08	0.000*	96.46 ± 3.87	0.000*	94.89 ± 4.21	0.000*
HVBHr (%)	68.97 ± 8.43	97.34 ± 4.85	0.000*	97.40 ± 6.11	0.000*	96.05 ± 5.21	0.000*	93.11 ± 3.22	0.000*
Cobb angle(°)	11.80 ± 1.44	2.46 ± 1.00	0.000*	2.72 ± 0.70	0.000*	3.03 ± 0.65	0.000*	4.34 ± 0.56	0.001*

*The difference between groups is statistically significant.*p* < 0.05

^a^findings before operation compared with those after reducion

^b^findings before operation compared with those 3 days after the operation

^c^findings before operation compared with those 3 months after the operation

^d^findings before operation compared with those 12 months after the operation

### Measurement results of the vertebral height and cobb angle

**T**he anterior height, middle height, AVBHr, MVBHr, and Cobb angle of the injured vertebral body in the observation after reduction, and 3 days, 3 months and 12 months post-operatively were compared with those of the injured vertebral body before operation. All parameters were significantly improved (*p* < 0.05).(Table 2) The anterior height, middle height, AVBHr, MVBHr, and Cobb angle of the injured vertebrae in the observation group after reduction, and 3 days, 3 months and 12 months post-operatively were significantly better than the results of the control group at the corresponding time points (*p* < 0.05) (Table 3).

**Table 3:** The anterior and middle heights, AVBHr, MVBHr and the Cobb angle of injured vertebrae pre- and post-operatively in the two groups

	Before the operation		Grobservation/Grcontrol *P*-value	After reduction		Grobservation/Grcontrol *P*-value	3 days after the operation		Grobservation/Grcontrol *P*-value	3 months after the operation		Grobservation/Grcontrol *P*-value	12 months after the operation		Grobservation/Grcontrol *P*-value
	observation group	control group		observation group	control group		observation group	control group		observation group	control group		observation group	control group	
anterior height (mm)	20.56 ± 3.74	20.70 ± 4.57	0.799	29.53 ± 2.53	28.32 ± 3.18	0.023*	29.88 ± 2.52	28.5 ± 5.09	0.019*	28.36 ± 1.93	27.03 ± 1.43	0.018*	27.96 ± 0.72	23.7 ± 0.66	0.001*
middle height (mm)	20.36 ± 4.20	19.36 ± 3.06	0.934	27.54 ± 1.00	26.43 ± 2.03	0.028*	27.70 ± 2.01	26.56 ± 4.56	0.021*	27.05 ± 2.45	24.12 ± 3.23	0.002*	26.83 ± 2.45	22.45 ± 2.57	0.001*
AVBH(%)	63.09 ± 8.91	63.76 ± 4.68	0.908	97.22 ± 7.38	93.31 ± 2.75	0.001*	97.54 ± 5.08	93.06 ± 3.57	0.001*	96.46 ± 3.87	85.64 ± 3.23	0.000*	94.89 ± 4.21	82.77 ± 4.97	0.000*
HVBHr(%)	68.97 ± 8.43	64.91 ± 4.84	0.059	97.34 ± 4.85	89.15 ± 3.57	0.000*	97.40 ± 6.11	89.13 ± 4.5	0.000*	96.05 ± 5.21	77.95 ± 5.67	0.000*	93.11 ± 3.22	74.8 ± 3.68	0.000*
Cobb angle(°)	11.80 ± 1.44	11.84 ± 1.78	0.693	2.46 ± 1.00	3.59 ± 1.03	0.002*	2.72 ± 0.70	3.65 ± 0.69	0.001*	3.03 ± 0.65	4.68 ± 0.23	0.001*	4.34 ± 0.56	7.89 ± 1.24	0.000*

*The difference between groups is statistically significant.*p* < 0.05

### Observation results of the VAS and GQOL-74 scores

At the last follow-up, the pain VAS score of the observation group was 1.3 ± 0.6 points and that of the control group was 2.9 ± 0.7 points; the GQOL-74 score of the observation group was 253.8 ± 7.8 points and that of the control group was 219.6 ± 7.2 points. The pain VAS score in the observation group was significantly better than that in the control group at the last follow-up (*p* < 0.05). The quality of life GQOL-74 score in the observation group was better than that in the control group at the last follow-up, the difference was not statistically significant (*p* > 0.05) (Table 4).

**Table 4:** The VAS and GQOL-74 scores at the last follow-up

	observation group	control group	*P*-value
VAS score at the last follow-up (point)	1.3 ± 0.6	2.9 ± 0.7	0.007*
GQOL-74 score at the last follow-up (point)	253.8 ± 7.8	219.6 ± 7.2	0.539

*The difference between groups is statistically significant.*p* < 0.05

### Observational results of bone grafting

**Fig. 4:**
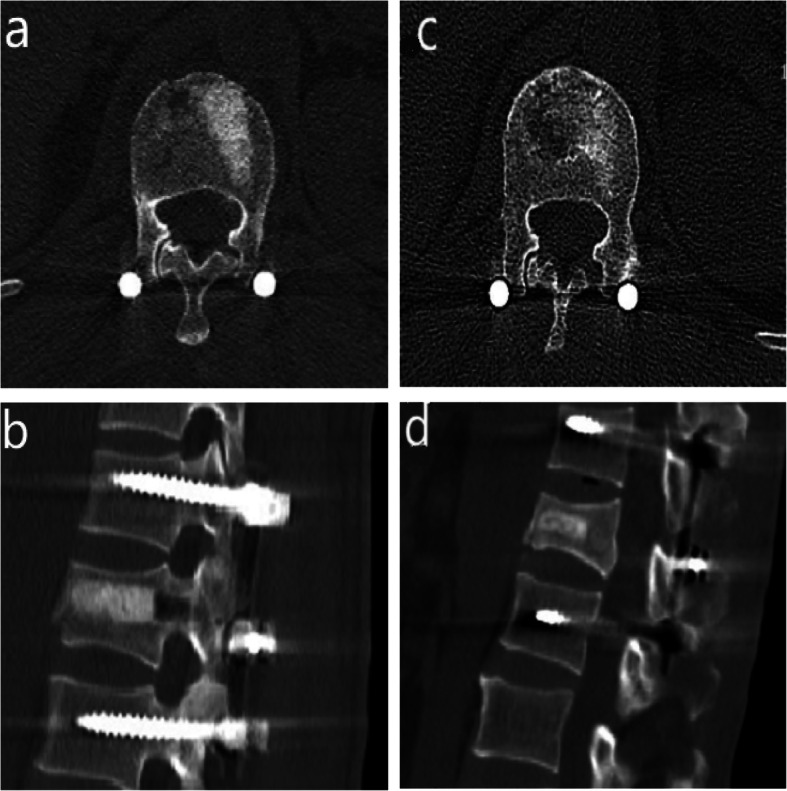
Coronal and sagittal CT images of the postoperative observation group. **a**,**b** Coronal and sagittal CT images of the observation group at 3 days after the operation. **c**,**d**:Coronal and sagittal CT images of the observation group at 12 months after the operation

In the observation group,4–9 g of allograft bone was used to fill the vertebral body (the average bone graft was 5.4 g) during the operation, and there were no complications, such as pedicle rupture and intravertebral haematoma. Three days after surgery, the allograft was evenly wedged in the anterior and medial columns..CT re-examination at 12 months after surgery revealed that most of the allogeneic bone used to fill the anterior and middle columns was absorbed, and no large bone defects were found. In addition, the trabecular structure could be seen in the cancellous bone, no obvious fracture line was seen, and the bone healed well (Fig. 4).

### Typical cases

**Fig. 5:**
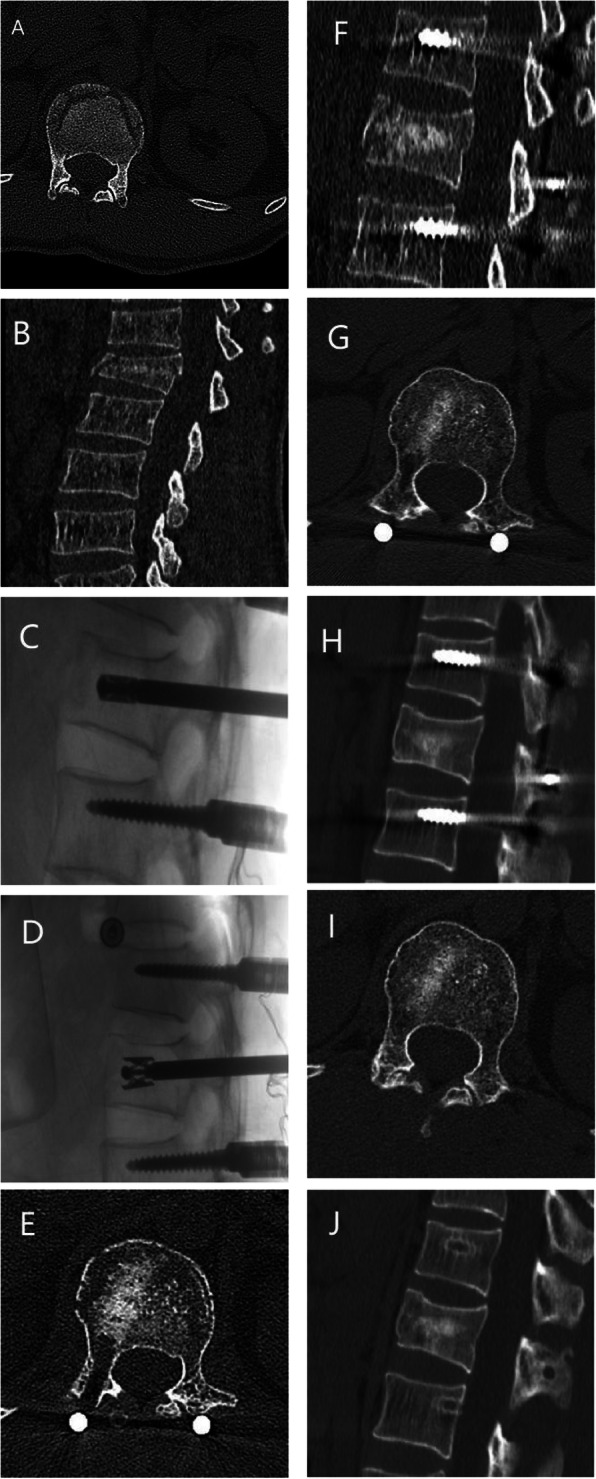
Imaging of a typical patient who underwent the novel transpedicular reducer procedure for reduction and bone grafting combined with pedicle screw fixation before, during and after the operation. **A**, **B**: Preoperative coronal and sagittal CT demonstrating a burst fracture of the vertebral body. **C**, **D**: During the operation, the new transpedicular reducer was used to reset the injured vertebra. **E**, **F**: Coronal and sagittal CT 3 days after the operation demonstrating that the allograft was evenly wedged into the anterior and medial columns. **G**, **H**: Coronal and sagittal CT 12 months after the operation. The allograft bone that filled the anterior and middle columns was partially absorbed. No defects were found, and the trabecular structure was visible in the cancellous bone. **I**, **J**: Coronal and sagittal CT after removal of the internal fixation

4.5.1 A 32-year-old male had a burst fracture of the L1 vertebra due to a fall and was treated with the novel transpedicular reducer for reduction and bone grafting combined with pedicle screw fixation as shown in Fig. 5.

**Fig. 6:**
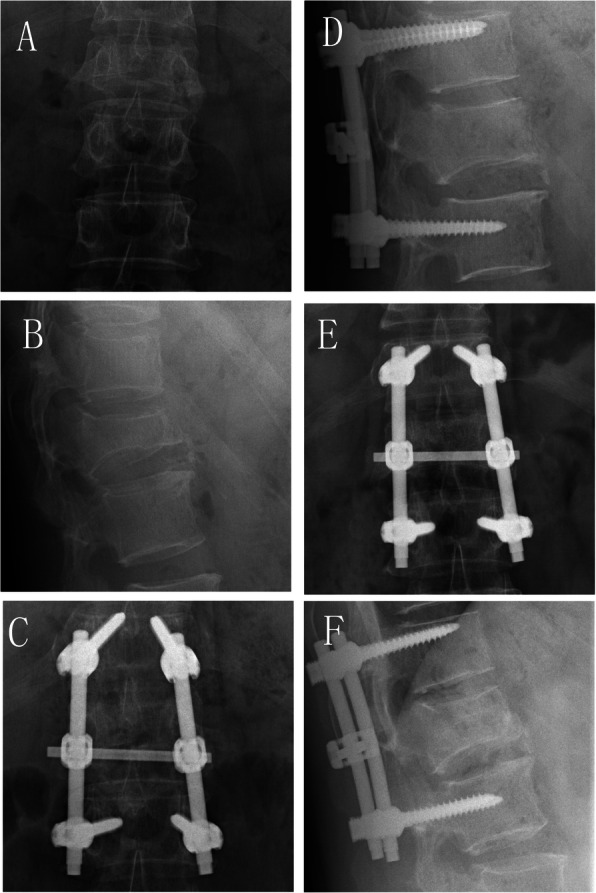
Imaging of a typical patient who underwent short-segment transpedicular screw fixation alone before and after the operation. **A**, **B**: Preoperative coronal and sagittal x-ray film demonstrating a burst fracture of the vertebral body. **C**, **D**: Coronal and sagittal x-ray film 3 days after the operation. **E**, **F**: Coronal and sagittal x-ray film 12 months after the operation

4.5.2 A 40-year-old male had a burst fracture of the L1 vertebra due to a fall and underwent short-segment transpedicular screw fixation alone as shown in Fig. 6.

## Discussion

TBFs are clinically common. Spinal fractures typically occur in the thoracolumbar segment. The transition from the less mobile thoracic spine and its associated ribs and sternum to the more mobile lumbar spine makes the thoracolumbar junction (T11-L2) a large area of biomechanical stress. The imaging features include rupture of the posterior wall of the vertebral body, retrograde entry of the posterior edge of the vertebral body into the lumen, a decrease in the height of the vertebral body and an increase in the distance between the pedicles. Holdsworth [17] believes that burst fractures cause damage to the anterior and middle columns and that the posterior column is often intact, which corresponds to a stable fracture. Denis [18] believes that damage to the middle column is an important indicator of stability. All thoracolumbar burst fractures have middle column damage, which corresponds to an unstable fracture. Conservative treatment includes bed rest and reduced posture and orthotics, which may help relieve pain for weeks or months. Conservative treatment of fractures has been shown to be useful in most stable fractures [11, 19–22] but not in all cases, and long-term bed rest is associated with an increased incidence of bedsores, pneumonia, venous thromboembolism, and even death [23]. Compared with nonsurgical methods, surgical treatment of thoracolumbar fractures does provide some advantages, especially for patients who cannot tolerate orthotics or plaster orthotics for several months, such as patients with multiple limb injuries, skin lesions, and obesity [12]. Therefore, in the present study, patients with contraindications to surgery were excluded, and surgical treatment was recommended for the remaining patients. Surgical decompression can also be more reliable and effective in removing the bone block protruding into the spinal canals, restoring neurological function and improving rehabilitation. In 1984, Denis et al. conducted a retrospective comparison between the surgical and nonsurgical treatment in 52 cases of blowout fractures without neurological defects and found that all patients treated with surgery had no relevant disability and returned to full-time work, whereas 25% of the patients treated without surgery were unable to return to full-time work [18]. In addition, neurological problems were reported in 17% of nonsurgical patients. Siebenga et al. concluded that surgical treatment not only offered better clinical outcomes but was also more cost-effective than nonsurgical treatment [24]. Two other large systematic evaluations [25, 26] demonstrated that early surgery for thoracolumbar fractures was associated with reduced complications and shorter hospital and ICU stays.

Surgical treatment of TBFs varies according to many factors. The shape of the fracture, the state of the nervous system, and the surgeon’s preference all play important roles in determining the surgical procedure. Short-segment pedicle screw fixation is now widely used but the acknowledged disadvantages of this procedure are early reduction failure and recurrent kyphosis [26, 27]. Because the cancellous bone in the vertebral body is compressed after a thoracolumbar burst fracture, often combined with endplate collapse, only the pedicle screw device is used to indirectly restore the fractured vertebra through distraction. The bone trabecula and spinal cord structure in the injured vertebrae are not completely reset, which will result in insufficient recovery of the depressed end plate [28]. Due to the existence of bone defects and voids in the vertebral body, an “eggshell-like” vertebral body is formed, which cannot provide sufficient support and stimulation for fractures within the vertebral body, resulting in insufficient support strength of the anterior and middle columns of the vertebral body. Therefore, bone healing is not complete. The “eggshell-like” vertebral body will further reduce the height of the injured vertebral body under the action of slight external force, which will eventually lead to the loss of the vertebral body height and even the failure of internal fixation [26, 29, 30]. In addition, due to the lack of ligament and annulus attachment, the collapsed central endplate cannot be fully repositioned, the intervertebral disc loses integrity, and gradual disc degeneration leads to loss of intervertebral disc height, stenosis of the intervertebral space, and increased kyphotic angle [31].

Therefore, placing these screws directly into the vertebrae without reduction may weaken the vertebrae, affecting subsequent restoration of the fracture and possibly leading to fracture displacement. Moreover, failure to perform targeted bone grafting and filling of the local “eggshell-like” cavity formed after the reduction of the injured vertebra will lead to further loss of vertebral height. Many scholars have further explored this concept and invented techniques such as SpineJack, Sky Bone Expansion System Kyphoplasty (SKP), Opti Mesh Vertebroplasty, Intravertebral Expandable Pillar (I-VEP) and Lantern bracket skeletal angioplasty to restore and support the shape and height of the fractured vertebral body, but there are still common shortcomings, namely: 1. All need to combine the existing bone cement technology or nail rod internal fixation system to achieve its application and 2. The techniques cannot provide a more uniform expansion and reduction force and the expansion height cannot be determined by itself.

Therefore, we designed a novel transpedicular reducer to treat compressibility and burst fractures. Our novel transpedicular reducer has the following characteristics: 1.Adopting the lever-regulating principle is labour-saving and convenient to implement; 2. It operates via the pedicle without breaking through the inner wall of the pedicle and will not cause nerve damage; 3.The contact surface of the stent surface with the bone tissue interface is increased to solve the problem posed by existing techniques in which the surface of the scaffold and bone tissue interface stress is too large; 4.Direct reduction of the injured vertebrae is more effective and 5.At the same time, it can provide vertebral space for bone grafting of injured vertebrae. Different from the above technology, our novel transpedicular reducer does not need to be combined with bone cement technology, which can provide uniform support, restore good controllability (according to the actual need) of the reset height and is easy to operate. According to our experimental research, although it is currently not possible for a single sample to reflect the independent reset effect of the novel transpedicular reducer, according to the data after restoration, it can be found that the anterior and middle heights of the injured vertebrae in the observation group recovered from the preoperative values of 20.56 ± 3.74 mm and 20.36 ± 4.20 mm to 29.53 ± 2.53 mm and 27.54 ± 1.00 mm, respectively, and the Cobb angle decreased from 11.80 ± 1.44° to 2.46 ± 1.00°, corresponding to statistically significant differences. Moreover, the anterior height, middle height, AVBHr and HVBHr of the injured vertebrae of the observation group were better than those of the control group, and the Cobb angle of the observation group was smaller than that of the control group, which was a statistically significant difference. Therefore, compared with short-segment transpedicular screw fixation alone, the novel transpedicular reducer has a certain reduction effect and can correct kyphotic deformity.

To solve the problem of “eggshell-like” vertebral bodies after performed with short-segment transpedicular screw fixation, several studies have demonstrated that reinforcing fractured vertebrae with bone cement such as polymethylacrylate can enhance fracture healing and prevent implant failure. However, PMMA has been reported to be associated with undesirable characteristics, such as a high temperature setting, possible damage to local nerve and vascular structures, inadequate bone fusion and a severe stiffness mismatch with bone, resulting in subsequent adjacent fractures and even vertebral restenosis [32]. Moreover, the leakage rate is so high (7–10%) that distal cement emboli enter the cardiac cavity and pulmonary system [33, 34].PMMA also cannot be replaced by biological tissue. As a result, scientists are now also looking for a new implant to minimize the incidence of complications. Cao et al. reported that allograft bone implantation in thoracolumbar fractures can effectively correct the Cobb angle and the height of the injured vertebral front and reduce the degree of the injured vertebral defect [35]. Therefore, we applied the novel transpedicular reducer and filled the damaged vertebral cavity with allograft bone through the bone graft channel, which effectively restored the vertebral bone structure and avoided leakage caused by the use of bone cement. CT results of the patients 12 months after the operation showed that most of the allogeneic bone used to fill in the anterior middle columns was absorbed, and no obvious defects were found. Trabecular structures were visible in the cancellous bone, and the bone healed well.

According to our research results at 3 days, 3 months and 12 months after surgery, the restoration effect of the novel transpedicular reducer for reduction and bone grafting combined with pedicle screw fixation was better than that of short-segment transpedicular screw fixation alone, and the difference was statistically significant (*p* < 0.05). According to the VAS and GQOL-74 scores, the clinical effect of the novel transpedicular reducer for reduction and bone grafting combined with pedicle screw fixation was better than that of short-segment transpedicular screw fixation alone, and the difference was statistically significant (*p* < 0.05). But the sample size of this study is relatively small. In the later study, we designed a large sample randomized controlled study, using the controlled variable method to compare the injured vertebrae at the same segment.

## Conclusion

The novel transpedicular reducer for reduction and bone grafting combined with pedicle screw fixation has a good therapeutic effect in patients with TBFs, which can effectively restore the injured vertebra and reduce complications and postoperative pain, thereby improving quality of life.

## Data Availability

All data generated or analysed during this study are included in this published article.
